# Individual cardiorespiratory fitness exercise prescription for older adults based on a back-propagation neural network

**DOI:** 10.3389/fpubh.2025.1546712

**Published:** 2025-04-30

**Authors:** Yiran Xiao, Chunyan Xu, Lantian Zhang, Xiaozhen Ding

**Affiliations:** ^1^Department of Sport Science Institute, Beijing Sport University, Beijing, China; ^2^Beijing Higher School Engineering Research Center of Sport Nutrition, Beijing Sport University, Beijing, China

**Keywords:** BP neural network, older adults, exercise prescription, cardiorespiratory fitness, experimental validation

## Abstract

**Introduction:**

To explore and develop a backpropagation neural network-based model for predicting and generating exercise prescriptions for improving cardiorespiratory fitness in older adults.

**Methods:**

The model is based on data from 68 screened studies. In addition, the model was validated with 64 older adults aged 60–79 years. The root mean square error (RMSE), mean absolute error (MAE) and coefficient of determination (R^2^) were used to evaluate the fitting and prediction effects of the model, and the hit rate was used to evaluate the prediction accuracy of the model.

**Results:**

The results showed that (1) The mean error ratios for predicting exercise intensity, time and period were 7% ± 12, −5% ± 9% and − 7% ± 14%, respectively, indicating that the estimates were in good agreement with the expected results. (2) Of the 61 subjects who completed the assigned program, cardiorespiratory fitness improved significantly compared with pre-exercise. Improvements ranged from 9.2–10% and 8.9–15.8% for female and male subjects. (3) In addition, 71 and 94% of subjects (43/61) showed cardiorespiratory improvement within plus or minus one standard deviation and plus or minus 1.96 times standard deviation.

**Discussion:**

A neural network-based model for exercise prescription for cardiorespiratory fitness improvement in older adults is feasible and effective.

## 1 Introduction

Cardiorespiratory fitness (CRF), a health-related component of physical fitness, refers to the ability of the circulatory and respiratory systems to supply oxygen to muscular systems during physical activity (1). Longitudinal studies have found that the decline of CRF over time range from 5 to 20% per decade from the age of 30 onwards (2), with older age groups demonstrating a steeper rate of decline (3, 4). CRF is associated with cardiovascular disease and all-cause mortality in both men and women (5). Furthermore, among older adults, satisfactory CRF is necessary for quality of life, functional preservation, and independence (2–6).

Among the methods to boost the CRF of older adults, exercise training based on a given exercise prescription is a proven strategy. Exercise prescription is a specific plan of fitness-related activities devised for a particular purpose (such as maintaining CRF), typically developed by a fitness or a rehabilitation specialist. It mainly includes frequency, intensity, type, and time of exercise (FITT), but also includes volume (V), and progress (P) (7–9).

The World Health Organization (WHO) recommends that older adults engage in at least 150–300 min of moderate-intensity aerobic physical exercise or at least 75–150 min of vigorous aerobic physical exercise per week; or an equivalent combination of moderate and vigorous exercise, for substantial CRF benefits (10). However, there is substantial heterogeneity in CRF response to a certain exercise prescription; some participants got a high improvement in CRF levels, some had no improvement with training (11, 12). In these studies, it may be limited by the high heterogeneity of dose parameters, participant characteristics, or both (13). The American College of Sports Medicine (ACSM) standpoint to ensure improved CRF in older adults recommends at least 30 min of moderate-intensity exercise at least 5 days per week or at least 20 min of high-intensity exercise at least 3 days per week. Additionally, previous studies have adopted a variety of methods to formulate an individualized CRF exercise prescription for older adults (14). For example, physicians or other healthcare providers may assess an older adult’s baseline and then formulate exercise prescriptions based on physician’s clinical experience or clinical guidelines. However, exercise prescriptions that are directly based on their work experience may not be applicable to all older adults, because each person’s age, sex, and physical condition are different, which may result in failure to achieve desired improvements (15). Therefore, to improve CRF in older adults, individualized exercise prescriptions should be formulated with consideration of factors such as age, sex, and physical condition.

To build an accurate and personalized predictive model for exercise prescription, comprehensive baseline data are essential for capturing individual variability. Neural Networks (NN), particularly the Back-Propagation Neural Network (BPNN), have emerged as powerful tools in health intervention studies due to their ability to approximate complex nonlinear relationships, resist noise interference, and adapt to heterogeneous systems (16–19). Recent advances in BPNN applications span fault detection (20) and medical diagnostics (17), demonstrating its versatility in handling noisy, high-dimensional data. Building on these foundations, our study extends BPNN to geriatric exercise prescription, addressing aging-specific physiological complexities. For instance, Beltrame et al. utilized NNs to predict exercise energy expenditure with high precision (21), while Frade et al. conclude that the CRF can be predicted by wearable technologies associated with NNs (22). However, existing studies predominantly focus on younger populations or single-parameter optimization, lacking dynamic modeling for multidimensional aging-related features. To address this gap, we hypothesize that a BPNN-based framework can dynamically integrate geriatric physiological complexity and generate individualized CRF-enhancing exercise prescriptions.

**Figure 1:**
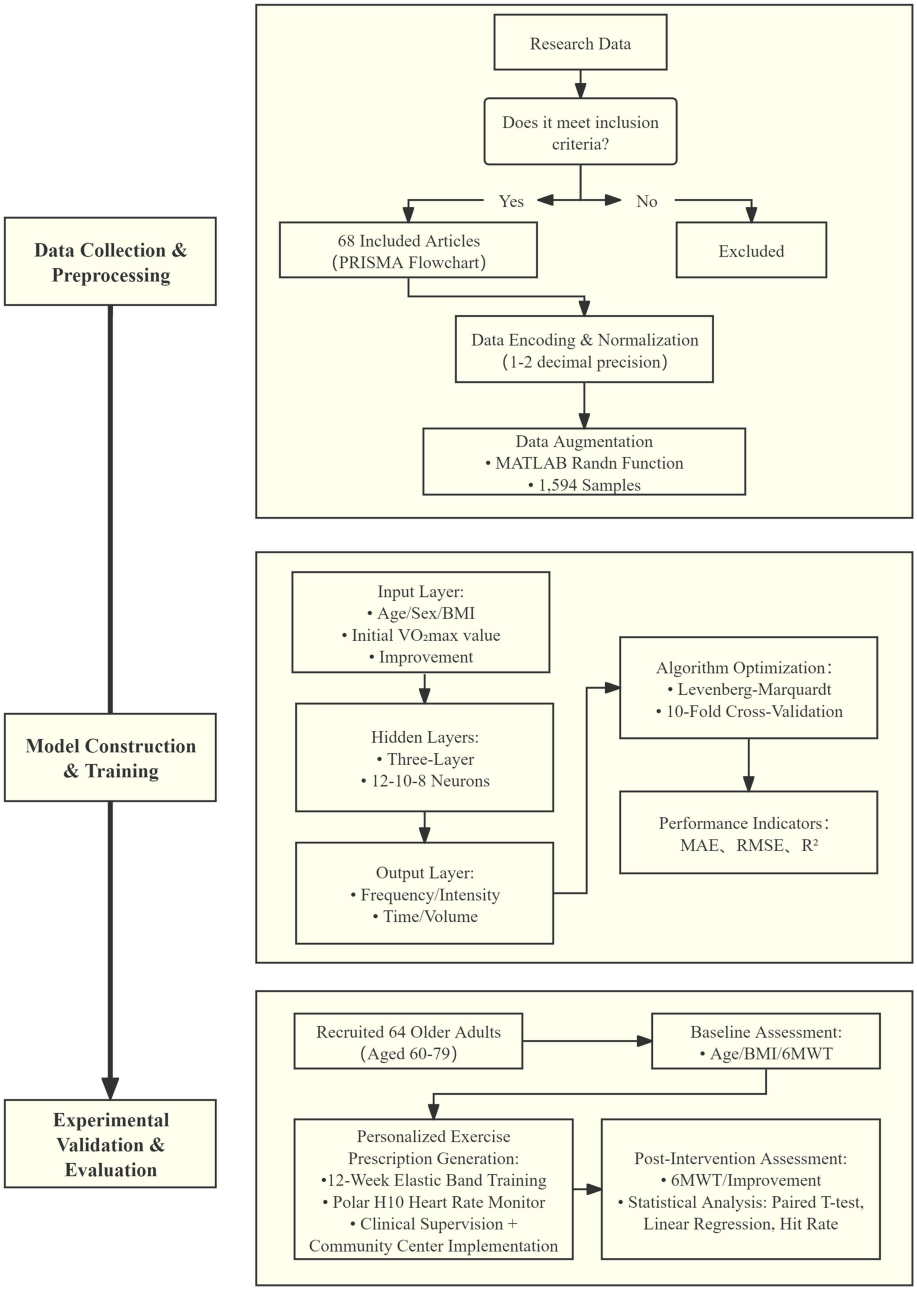
The research methodology.

Our study employed a mixed-methods approach, combining data from a systematic literature review to develop the model and experimental data to validate it. This dual-source strategy leverages historical intervention patterns and real-world heterogeneity, balancing clinical feasibility with personalized adaptability. The model aims to optimize exercise prescription for older adults, promoting their physical health and functional independence. The research methodology of this study is depicted in Figure 1.

## 2 Methods

### 2.1 Data collection

We collected and analyzed previous experimental research aimed at improving the CRF of older adults. Databases including PubMed, EBSCO, Web of Science and CNKI were searched for research published from 1989 to 2021 (last search in June 2021). A combination of keywords related to the three topics of this study—older adults, exercise intervention, and cardiorespiratory fitness—was used as search terms, as listed in Table 1.

**Table 1:** Keywords of exercise intervention on improving cardiopulmonary fitness in the older adults.

Search words	Older adults	Exercise intervention	Cardiorespiratory fitness
Key words	Older adult	Exercise	Cardiovascular fitness
	Geriatric	Training	Cardiopulmonary
	Aging	Physical activity	Cardiorespiratory fitness
	Aged	Aerobic training	VO_2_max
		Combined training	

**Figure 2:**
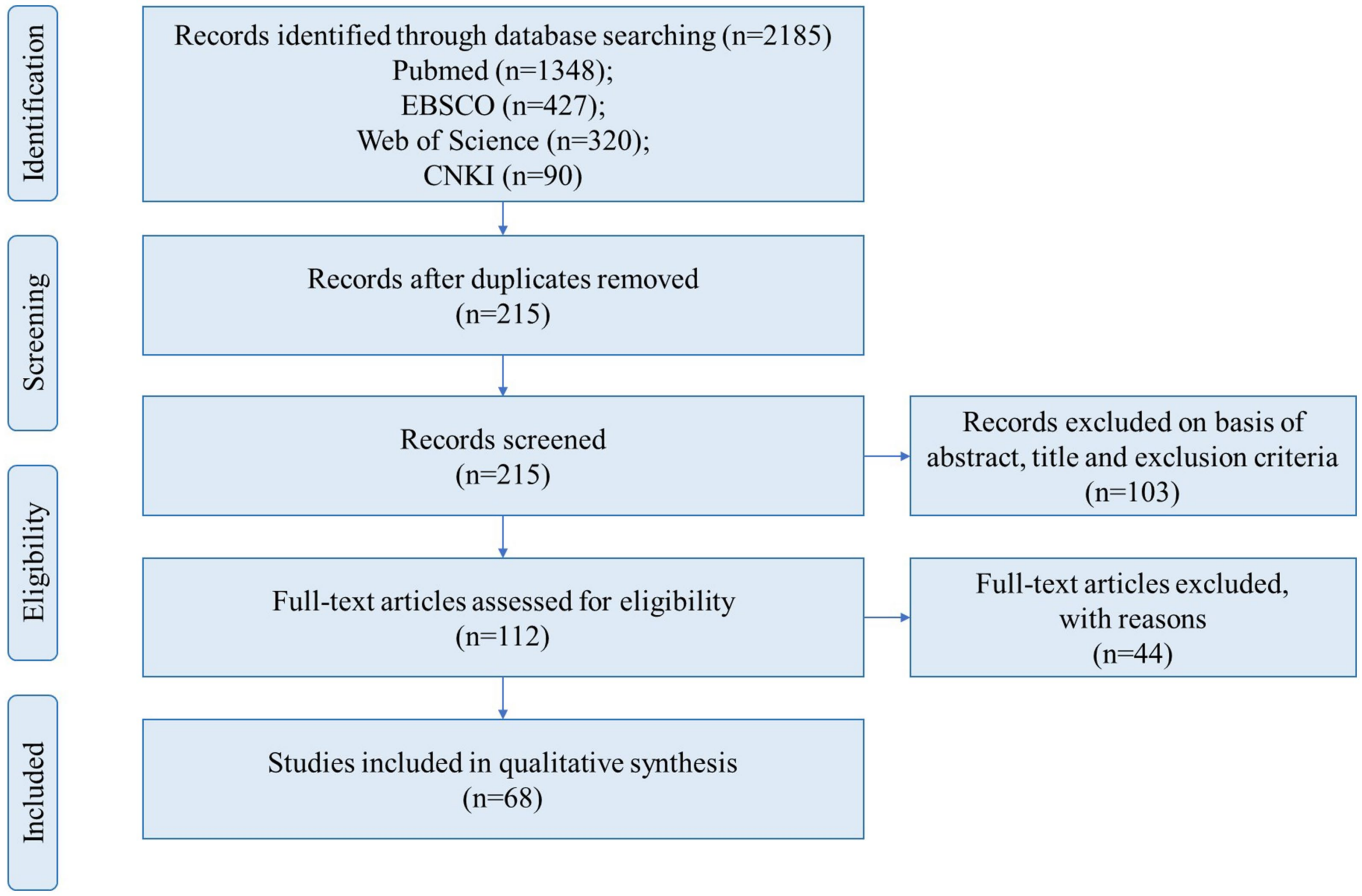
PRISMA flow-chart.

Additional synonymous search terms were included, guided by the articles retrieved during the literature search. The inclusion criteria covered studies written in English or Chinese using a randomized controlled trial or a self-control trial experimental design with subjects aged 60 years or older. Studies needed to focus on exercise interventions for older adults, outcome indicators all need to measure Maximal Oxygen Uptake (VO_2_max), and studies with or without a control group were acceptable. Conference papers, poor quality papers, and studies of exercise combined with nutritional supplementation would be excluded. All steps of the process followed the recommendations of the PRISMA Flow Diagram (23), as presented in Figure 2. After screening, a total of 68 articles (involving 1,594 subjects) met the above inclusion criteria and were subjected to data processing (24–91). Characteristics of the literature of the included studies are presented in Supplementary Table S1. The quality of the included studies was evaluated using the Physiotherapy Evidence Database (PEDro) scale (92), with results detailed in Supplementary Table S2.

### 2.2 Data processing

#### 2.2.1 Data encoding

Research by Luan et al. (15) identified age and sex as important factors influencing CRF. This evidence strongly supports our decision to include these variables in the model construction. In each research, we summarized the basic information include age, Body Mass Index (BMI), VO_2_max initial value (VO_2_max pre), improvement and sex, and exercise prescription elements of the research’s subjects, include exercise frequency, intensity, time and volume.

To prepare the available data as input and output for training the model, we encoded this basic information and exercise prescription elements to make them suitable for incorporation into the model program. Values for certain information are retained to one decimal place (e.g., age, BMI and VO_2_max pre). We calculate the VO_2_max improvement and keep two decimal places (VO_2_max final value = VO_2_max post).

When utilizing the trained BPNN, both input sets should be encoded, but also the output data sets should be encoded. Exercise prescription elements are established as our output sets. The encoding of the output dataset is the same as the input set. For the exercise intensity set, we extract the average value of the exercise intensity range, convert it to a decimal and subtract 0.5 [taking 50% Heart Rate Reserve (HRR) exercise intensity as 0], such as a study’s exercise intensity is 60% HRR, in our exercise intensity set is 0.1. In the case of exercise time and volume set, we keep two decimal places.

#### 2.2.2 Data augmentation

To increase the sample size reasonably, we chose age, BMI, VO_2_max initial value as the parameters for data augmentation (mean ± standard deviation) based on the collected studies as these parameters showed greater variability in the dataset and were closely related to the study objectives. Additionally, sex and VO_2_max improvement were set as fixed valued because they are relatively stable and have less impact on model performance (93).

Data augmentation is performed through the Randn function on MATLAB (R2021a; MathWorks, Natick, MA, United States). These generated numbers have passed various statistical tests of randomness and independence, and their calculation can be repeated for testing or diagnostic purposes (94).

We simulated the basic information of each subject in each study, with each subject’s information generated in a 1*3 matrix format, we simulated 1,594 groups of sample data.

### 2.3 Back-propagation neural network model

#### 2.3.1 Input and output layer setting

The number of neurons in the input unit has a direct impact on the prediction outcome. We adopted a multi-input multi-output (MIMO) architecture:

Input parameters (5 dimensions): Age, Sex, BMI, VO_2_max initial value, and improvement.

Output parameters (4 dimensions): Exercise frequency (days/week), intensity (%HRR), time (minutes), and volume (weeks).

Continuous variables (e.g., age, BMI) were standardized according to data coding method. Categorical variables (sex) were coded using One-Hot Encoded (0 = female, 1 = male) to avoid numerical bias in weight updates. To achieve a precise model, prior to training the BPNN, we need to set the parameters of the neural network, the selection of neuron transfers functions, and neural network training algorithm and errors, etc. (93).

#### 2.3.2 Model algorithm selection

The traditional BPNN suffers from slow convergence and local minima trapping. The Levenberg–Marquardt (LM) algorithm was implemented to enhance weight updates:


(1) $$\Delta w={\left(J^{T}J+\lambda I\right)}^{-1}J^{T}e$$

in Equation 1 *J* is the Jacobian matrix of errors to weights, *λ* is the damping factor, and *e* is the error vector. LM dynamically adjusts *λ*:

Decrease *λ* when errors decline, approximating the Gauss-Newton method for faster convergence.

Increase *λ* when errors rise, reverting to gradient descent for stability.

Compared to standard gradient descent (manual learning rate tuning), LM leverages second-order derivative information for adaptive step size optimization, particularly effective for medium-scale networks (<1,000 parameters) (95). Despite higher memory demands (storing *J*), matrix optimization in MATLAB Deep Learning Toolbox.

#### 2.3.3 Model layers selection

In BPNN model, the parameter setup is crucial, with the number of nodes in the hidden layer and neurons in the input set unit being key variables. It has been established that a BPNN with a single hidden layer and sufficient neurons can realize any nonlinear mapping (90). However, if the sample number is large, a network with one hidden layer fails to achieve an accurate function, and the calculation efficiency decreases significantly (96). To create an accurate model, it is necessary to determine the optimal number of hidden layers, with the number of neurons in each hidden layer given by Equation 2.


(2) $$n_{1}=\sqrt{n+m}+a$$

where n_1_ is the number of hidden neurons, n is the number of input units, m is the number of output units, a is a constant between [1,10] (97). According to the empirical formula, the neuron range was set to 4–14.

**Figure 3:**
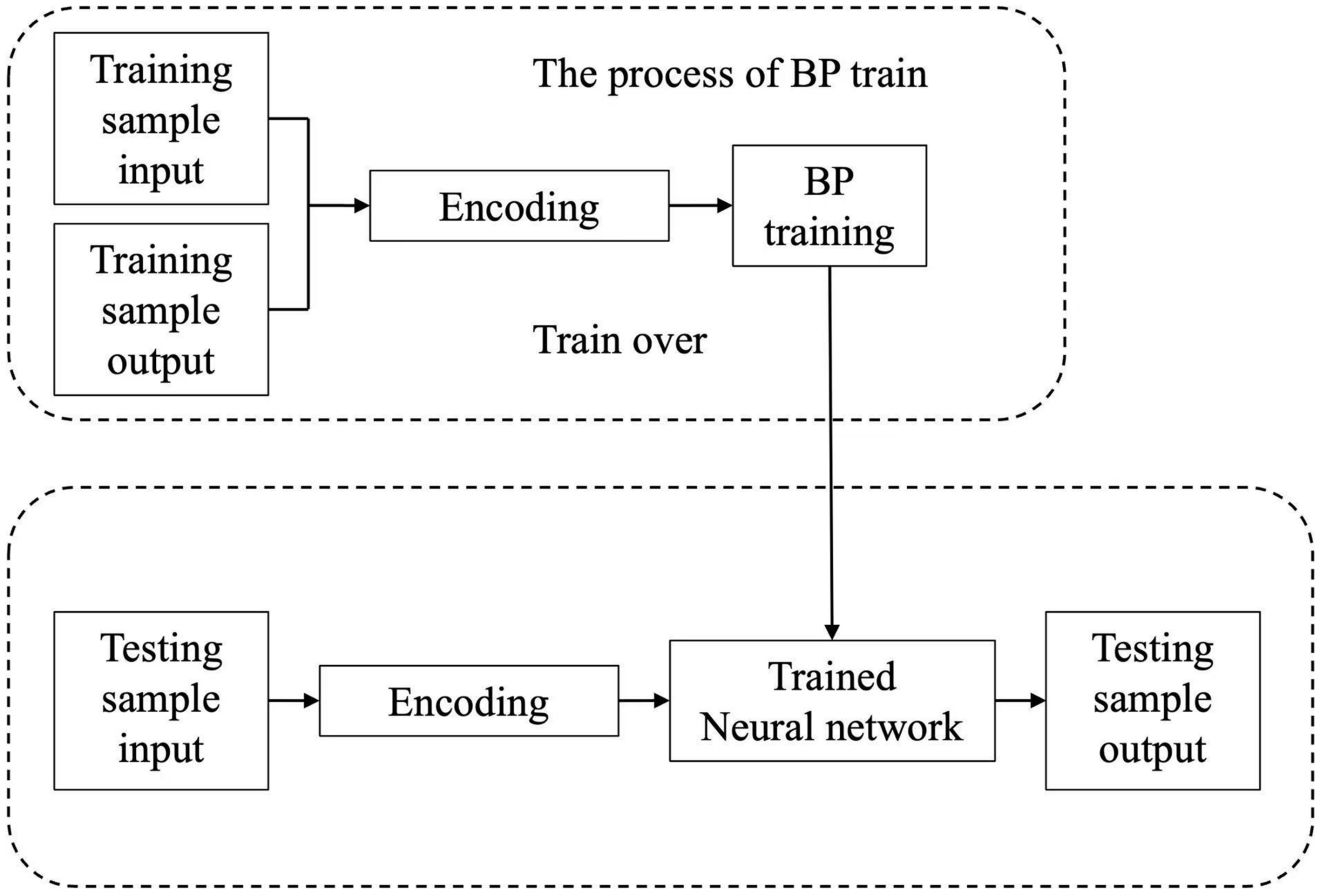
BPNN training and testing process.

Grid search across 1–7 hidden layers revealed: Single hidden layer (12 neurons): RMSE = 1.62, faster training but prone to underfitting. Three hidden layers (12–10-8 neurons): RMSE = 1.44 with minimal validation error fluctuation. Deep layers enabled hierarchical feature extraction: baseline traits (age/BMI) in the first layer, nonlinear intensity thresholds in intermediate layers, and exercise prescription integration in the final layer. The BPNN processing is depicted in Figure 3.

### 2.4 Model training and validation

#### 2.4.1 Model tuning and regularization strategies

To enhance model performance, a systematic hyperparameter tuning process was conducted. Key parameters included: (i) Learning Rate (*η*): Governs the step size during gradient descent. Tested values: {0.001,0.01,0.1}. (ii) Momentum Factor (*β*): Accelerates convergence by smoothing weight updates. Tested values: {0.5,0.7,0.9}. (iii)L2-Regularization Coefficient (*α*): Penalizes large weights to prevent overfitting. Tested values: {0,0.001,0.01}. The optimal combination was determined via grid search, minimizing validation Root Mean Squared Error (RMSE).

To balance model complexity and generalizability, L2 regularization was integrated into the loss function:


(3) $$L=MSE+\alpha \overset{w}{\underset{j=1}{\sum }}w_{j}^{2}$$

where *W* is the total number of weights. Early stopping monitored validation loss, terminating training if no improvement occurred for 10 consecutive epochs, effectively preventing overfitting (98).

The LM algorithm implemented dynamic damping factor adjustment (initial *λ* = 0.01) with finite-difference Jacobian approximation (*Δ* = 10^−5^) (95), synergized with the L2-regularized Mean Squared Error (MSE) loss in Equation (3) the default choice in MATLAB’s LM implementation (trainlm) for hardware-accelerated stability. This integrated design, validated by Luttmann et al. (99), inherently unifies adaptive step control and weight regularization through shared matrix optimization routines, demonstrating proven noise resilience in physiological signal regression. Combined with early stopping (10-epoch validation patience) (98), the framework establishes a robust defense against overfitting while maintaining computational efficiency.

#### 2.4.2 Model training

This study needs to determine the appropriate division of the data set to train the model and test the model. The dataset was partitioned into: (i) Training set (80%): For weight updates. (ii) Validation set (10%): For hyperparameter tuning and early stopping. (iii) Test set (10%): For final performance evaluation.

Cross-validation, a statistical method, is commonly used in applied machine learning to compare and select a model for a given predictive modeling problem and usually produces skill estimates with lower bias than other methods.

To ensure robust performance estimation, a nested cross-validation framework was employed: (i) Outer loop: 10 folds for train-test splits. (ii) Inner Loop: 10folds for hyperparameter optimization within each training subset. For each outer fold: Model weights were reinitialized to avoid bias. Training Leveraged LM optimization with the selected hyperparameters. Predictions on the test fold were aggregated to compute global metrics. Final metrics were averaged across all folds. This approach reduced sampling bias and provided a conservative estimate of generalization error.

#### 2.4.3 Model validation

##### 2.4.3.1 Evaluation indicators

To assess the accuracy of the model, the following evaluation indicators were selected: (i) Mean Absolute Error (MAE), (ii) RMSE, (iii) R-squared (R^2^), (iv) MSE, (v) Correlation Coefficient (R) and (vi) Error Ratios.

The MAE and RMSE were calculated to provide a measure of the average magnitude of the errors in the predictions. A value closer to 0 indicates higher accuracy of the model. The R^2^ was evaluated to assess the proportion of the variance in the dependent variable that is predictable from the independent variable(s). A value closer to 1 indicates a better fit of the model to the data. The model’s performance is quantified by the MSE, which measures the average squared difference between the predicted and actual values—with a lower MSE indicating better performance—and by the R, which assesses the strength and direction of the linear relationship between the predicted and actual values, with *r* = 1 signifying a perfect positive linear relationship. The error ratios were calculated relative deviations (%) for prescription elements.

##### 2.4.3.2 Experimental validation

**Figure 4:**
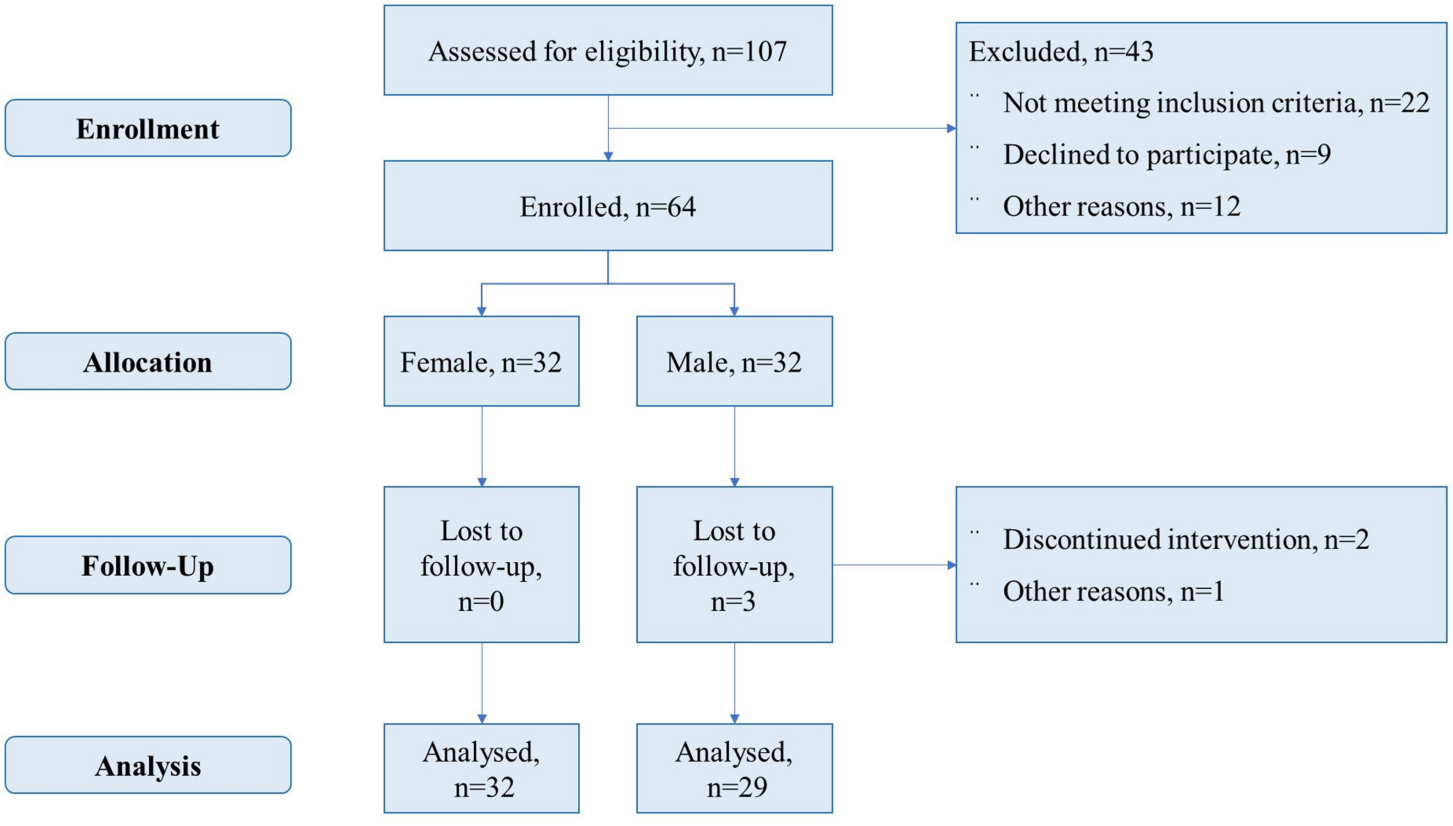
Flow through study.

In total, 64 older adults, aged 60–79, were recruited from a community center in an urban area of Beijing to validate the practical application of the model (Figure 4). These subjects often met for social activities at the center (although they did not engage in physical exercise), were undergoing regular health check-ups in primary healthcare, and were invited to participate in the research. The inclusion criteria were as follows: (i) aged 60–79, for both sexes; (ii) self-reliant; (iii) having adequate vision and hearing to participate in the intervention; and (iv) free from major physical and/or cognitive disease/disability that could affect participation. All subjects completed health risk screening and fully understood the purpose of the study, the trial process, and subject rights and responsibilities before their inclusion in the trial, and voluntarily signed an informed consent form. Participants engaged in a similar intervention during the same follow-up period were excluded. The research received approval from the Ethics Committee of Beijing Sport University (Ratification number: 2018018H), and all participants signed an informed consent form.

Supervised aerobic exercises were conducted at the Community Center’s Activity Plaza, using exercise equipment in the form of elastic bands, and participants performed aerobic exercise with elastic bands under the guidance of instructors. Exercise hours are during regular business hours, excluding legal holidays. Participants carried out their individualized program at designated times under the supervision of a staff-to-participant ratio of one to five. The staff included athletic trainers, as well as graduate and undergraduate students specializing in exercise science. Participants were also encouraged to exercise at home to reach their exercise targets. The degree of improvement in the model was set with reference to data from the literature used for modeling. Literature findings in the literature showed that after 12-weeks of exercise intervention, CRF improved by an average of 10% in older adults’ subjects (25, 26, 32, 41, 53, 84), so the degree of improvement was set to 10% for all subjects in the model. This approach aimed to enhance the model’s realism and the accuracy and reliability of its prediction. The individualized exercise prescription (frequency, intensity, time and volume) for each subject to improve 10% CRF was obtained from the model by entering the subject’s basic information. Aerobics was chosen for the intervention, with exercise intensity gradually increasing to match the individual intensity as prescribed by the model. The Polar H10 heart rate monitoring system (ECG single-lead, sample rate 130 Hz, Polar, Finland) was used to record a person’s heart rate during exercise, allowing for adjustments to the exercise intensity. A 6-min walk test (6MWT) was performed before and after the intervention to evaluate improvements in CRF. Ultimately, 61 subjects completed the study, resulting in a dropout rate of 4.7%. The basic characteristics of the subjects are presented in Table 2. The subjects’ estimated VO_2_peak (E-VO_2_peak) was based on the 6MWT results (100).

**Table 2:** The basic characteristics of the subjects.

Characteristics	Female (*n* = 32)	Male (*n* = 29)
Age (years)	68.5 ± 4.2	69.2 ± 6.1
Height (cm)	156.5 ± 5.2	166.1 ± 6.0
Weight (kg)	60.4 ± 9.5	67.8 ± 8.3
BMI (kg/m^2^)	24.6 ± 3.4	24.5 ± 2.4
6MWT (m)	527.1 ± 70.8	531.5 ± 89.3
Pre-E-VO_2_peak (ml kg^−1^ min^−1^)	11.9 ± 2.2	12.2 ± 2.6
Improvement (%)	10	10

The following statistical methods were employed in this study: Linear regression analysis was used to evaluate the relationship between expected-E-VO₂peak and post-E-VO₂peak. A paired sample t-test was conducted to compare pre-E-VO₂peak and post-E-VO₂peak values. Additionally, the “hit rate” (predictive accuracy) was calculated to assess the model’s predictive goals and to reveal the direction and magnitude of model bias through Bland–Altman analysis (101).

## 3 Results

### 3.1 Model design

**Figure 5:**
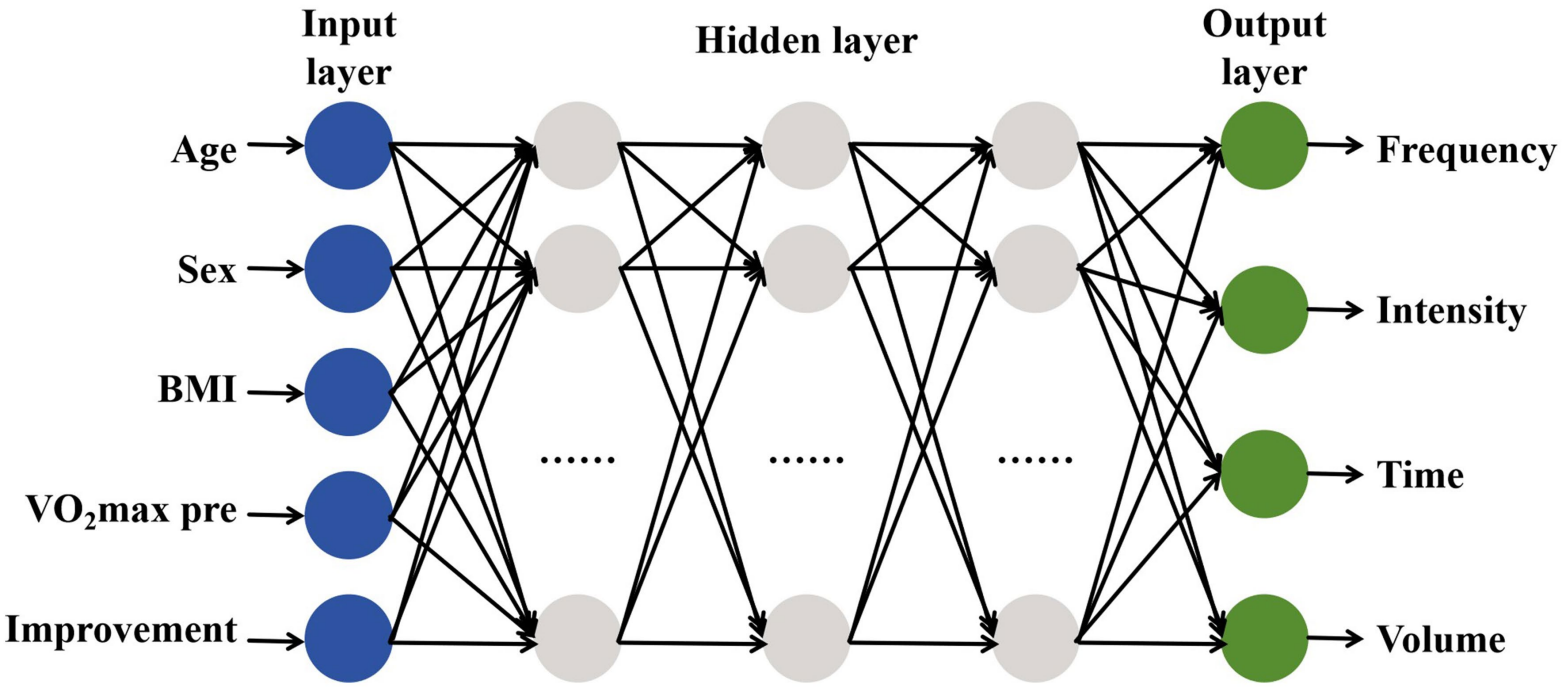
The structure of BPNN.

We selected 68 articles and 1,594 datasets as the dataset. We divided the dataset into 10 segments, using eight of them (*N* = 1,275) for weight updates, one (*N* = 159) for hyperparameter tuning and early stopping and the remaining one (*N* = 159) for final performance evaluation. The structure of the model incorporated a five-dimensions input layer, three hidden layers with dimensions 12–10-8, and a four-dimensions output layer (Figure 5). Table 3 shows the hyperparameter optimization results of the BPNN, and Table 4 shows the results of cross-validation.

**Table 3:** Hyperparameter optimization results of the BPNN.

Basic information network parameter	Parameter setting
Learning Rate (η)	0.01
Error Precision	10^−7^
Momentum Factor (β)	0.9
L2 Regularization Coefficient (α)	0.001
Display Interval	25
Maximum Number of Training Epochs	1,000

**Table 4:** Comparison of error indicators of each round of cross-validation.

Round	RMSE	MAE	R^2^
1	1.4190	1.6433	0.9946
2	1.3964	1.4312	0.9947
3	1.4777	1.6964	0.9941
4	1.6234	1.3363	0.9930
5	1.5250	1.4587	0.9938
6	1.3549	1.4226	0.9951
7	1.2620	1.2711	0.9957
8	1.5960	1.8182	0.9932
9	1.2366	1.5394	0.9959
10	1.4918	1.5887	0.9940
Mean	1.4383	1.5206	0.9944
Best	1.2366	1.5394	0.9959

Confirms the predictive superiority of our model. By comparing the mean and best values of RMSE and MAE (1.4383 vs. 1.5206) and (1.2366 vs. 1.5394). The R^2^ values exceeded 0.99, further proves the model’s predictive accuracy. Comparison with previous studies. Dai et al. (102) developed a prediction model for student fitness assessment using BPNN. It collected 332 datasets and reported a MAE of approximately 2.649 and an RMSE of 3.032. Likewise, in another study, Lin et al. (103) focused on predicting the brain age of healthy older adults using an improved BPNN based on the LM algorithm. It collected data from 112 subjects and achieved an average MAE of 6.14 and RMSE of 6.77 for brain age prediction. The above results imply that the present model can accurately predict the exercise intensity, time and volume.

**Figure 6:**
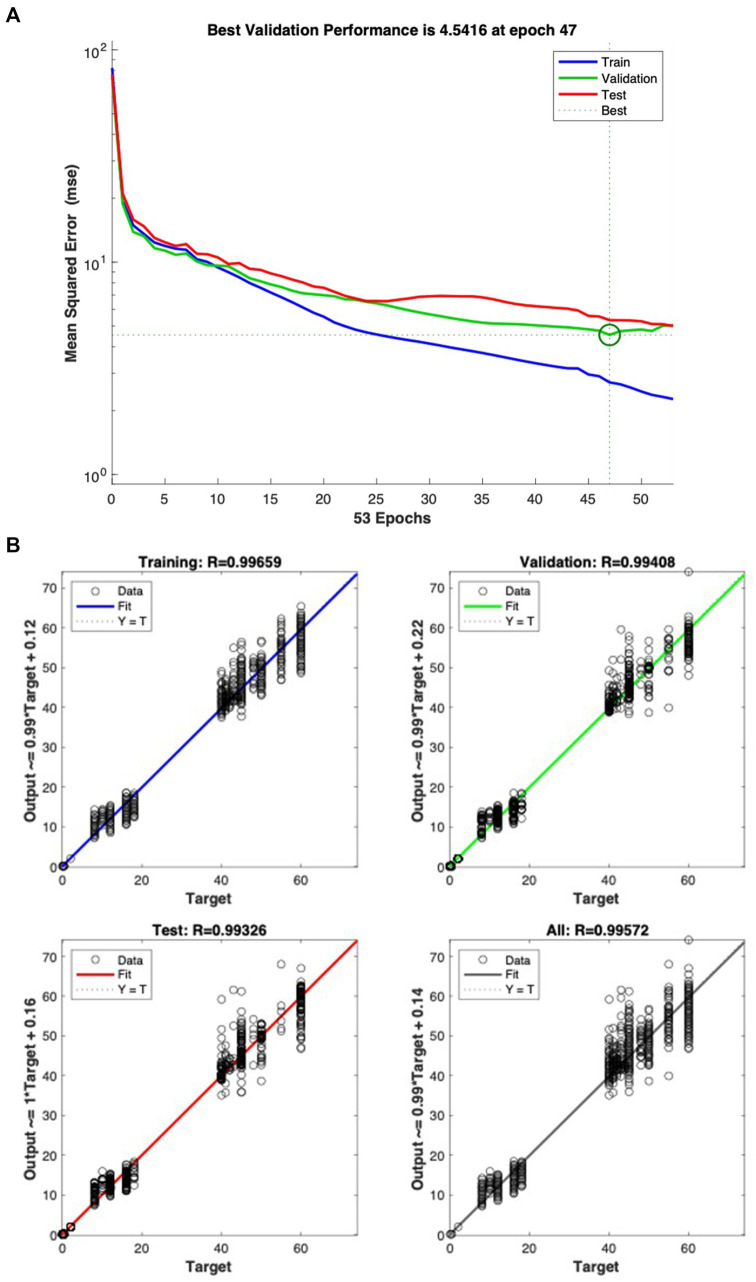
**(A)** Fitting regression diagram of LM-BPNN model. **(B)** Training error diagram of LM-BPNN.

The study results showed that the MSE of the predicted model was 4.5416 in Figure 6A, with R values for the training set, validation set, test set, and total being 0.99659, 0.99408, 0.99326, and 0.99572, respectively in Figure 6B. This result is consistent with Xie et al.’s study (104), which proposed a BPNN-based approach for developing postgraduate students’ mental health status diagnostic model. Their model, trained on 461 datasets, had an MSE of 13.1279 and R values of 0.98448, 0.97373, 0.98128 and 0.98273 for the training, validation, test and total sets, respectively. This indicated that our BPNN model has higher accuracy in predicting exercise intensity, time, and volume. Figure 6A illustrates the training error diagram of the LM-BPNN, with the horizontal axis representing the training epoch and the vertical axis representing the MSE on the dataset. Figure 6B shows the regression fitting values R for the training, validation, testing and overall sets, respectively.

**Figure 7:**
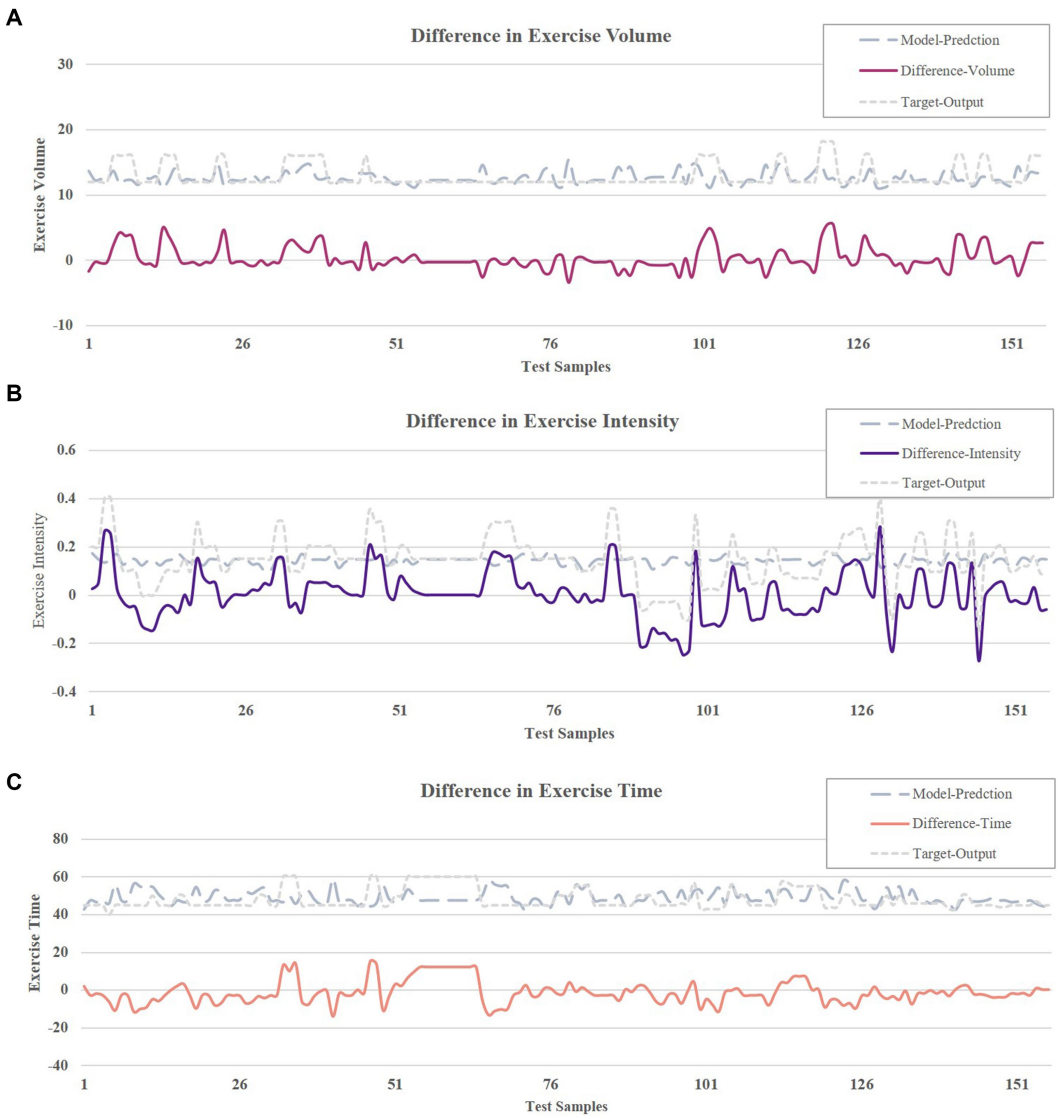
**(A)** Output difference in exercise intensity. **(B)** Output difference in exercise time. **(C)** Output difference in exercise volume.

Following the training of the BPNN model was, we tested its prediction ability Figures 7A–C illustrate the difference between the target output versus model predictions for the data used for model testing (*N* = 159). The average difference is 0.003 ± 0.099 for exercise intensity, −1.127 ± 6.094 for exercise time and 0.314 ± 1.750 for exercise volume. The predicted values closely matched the target output values, demonstrating impressive prediction accuracy.

**Figure 8:**
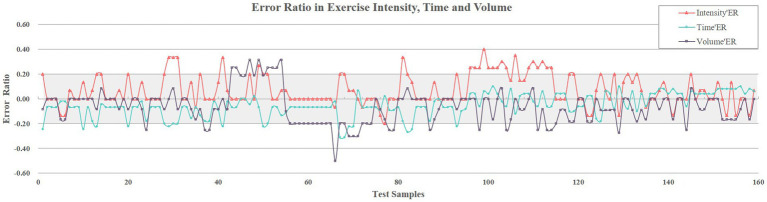
Error ratio in exercise intensity, time and volume.

Further error analysis using error ratios for elements revealed that Figure 8 systematically quantifies the three elements error ratios for the 159 test samples, with the gray shaded band (−20 to 20%) delineating the acceptability threshold. Specifically, 86% of the intensity errors (red), 91% of the time errors (blue), and 84% of the volume errors (black) fall within this range. Quantitative results showed average error ratios of 7 ± 12, −5% ± 9% and − 7% ± 14% respectively, with most error ratios below 20% tolerance threshold. These results confirm the operational reliability of the model considering the errors inherent in the coding/augmenting process of exercise prescriptions.

### 3.2 Experimental validation results

**Figure 9:**
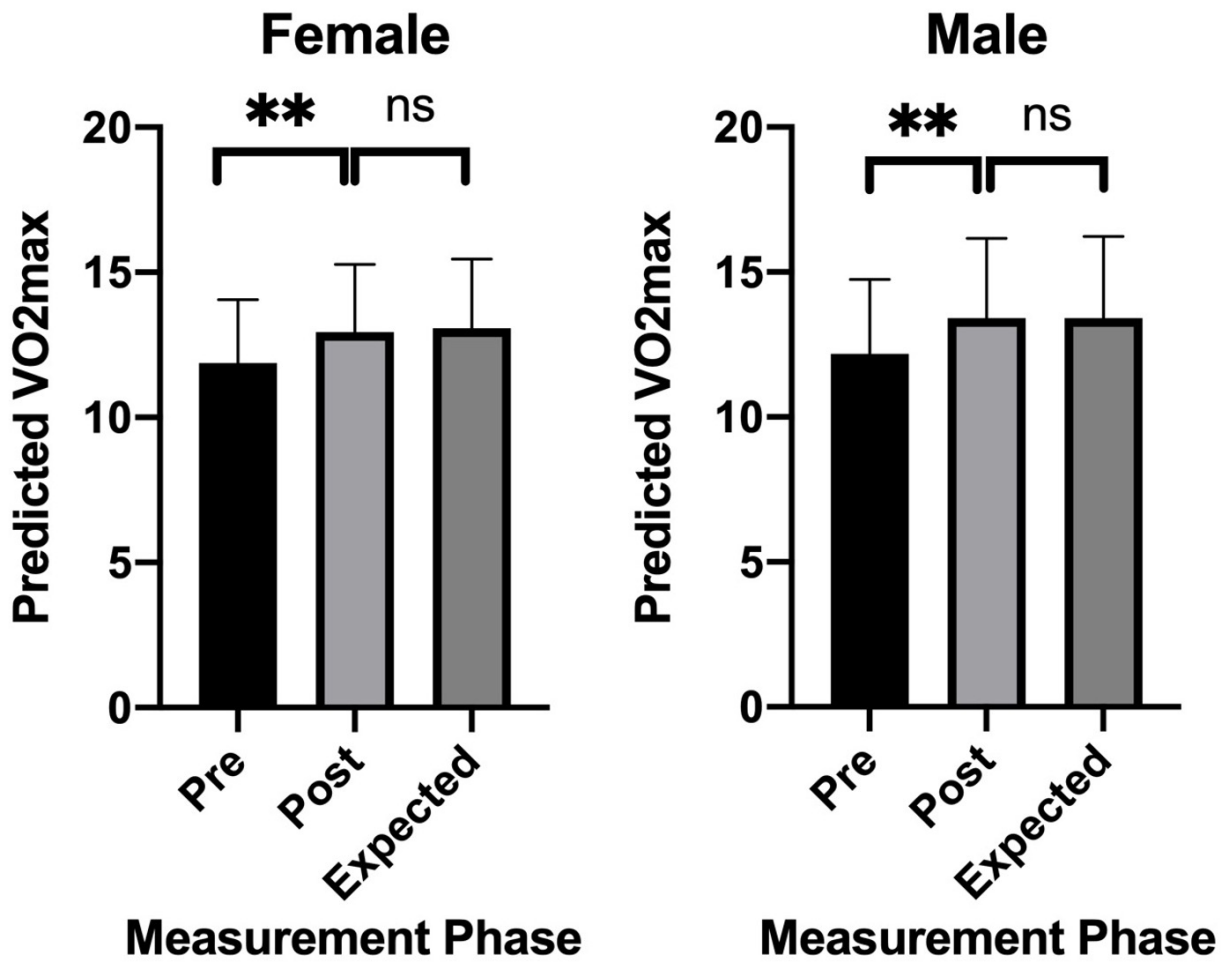
Difference in pre-E-VO2peak, post-E-VO_2_peak and expected-E-VO_2_peak.

The model-generated exercise prescriptions (Table 5), recommended twice-weekly sessions at 64% HRR, averaging 48 min (45–55 min) over 12 weeks. Post-intervention, significant improvements in E-VO_2_peak were measured again using the 6MWT, with females and males achieving 8.9 and 10.0% gains, respectively (*p* < 0.001; Figure 9). During the exercise intervention, all subjects showed well adaptability and tolerance with no serious side effects or sports injuries, proving the safety of the exercise intervention program.

**Table 5:** Model-generated exercise prescriptions for subjects.

Exercise Prescriptions	Female (*n* = 32)	Male (*n* = 29)
Frequency (d/w)	2	2
Intensity (%HRR)	65.0 ± 2.6 (60-68)	64.1 ± 4.1 (56-76)
Time (min)	46.9 ± 3.3 (39-55)	48.7 ± 3.2 (42-56)
Volume (w)	12.2 ± 1.0 (10-15)	12.3 ± 0.4 (12-14)

**Figure 10:**
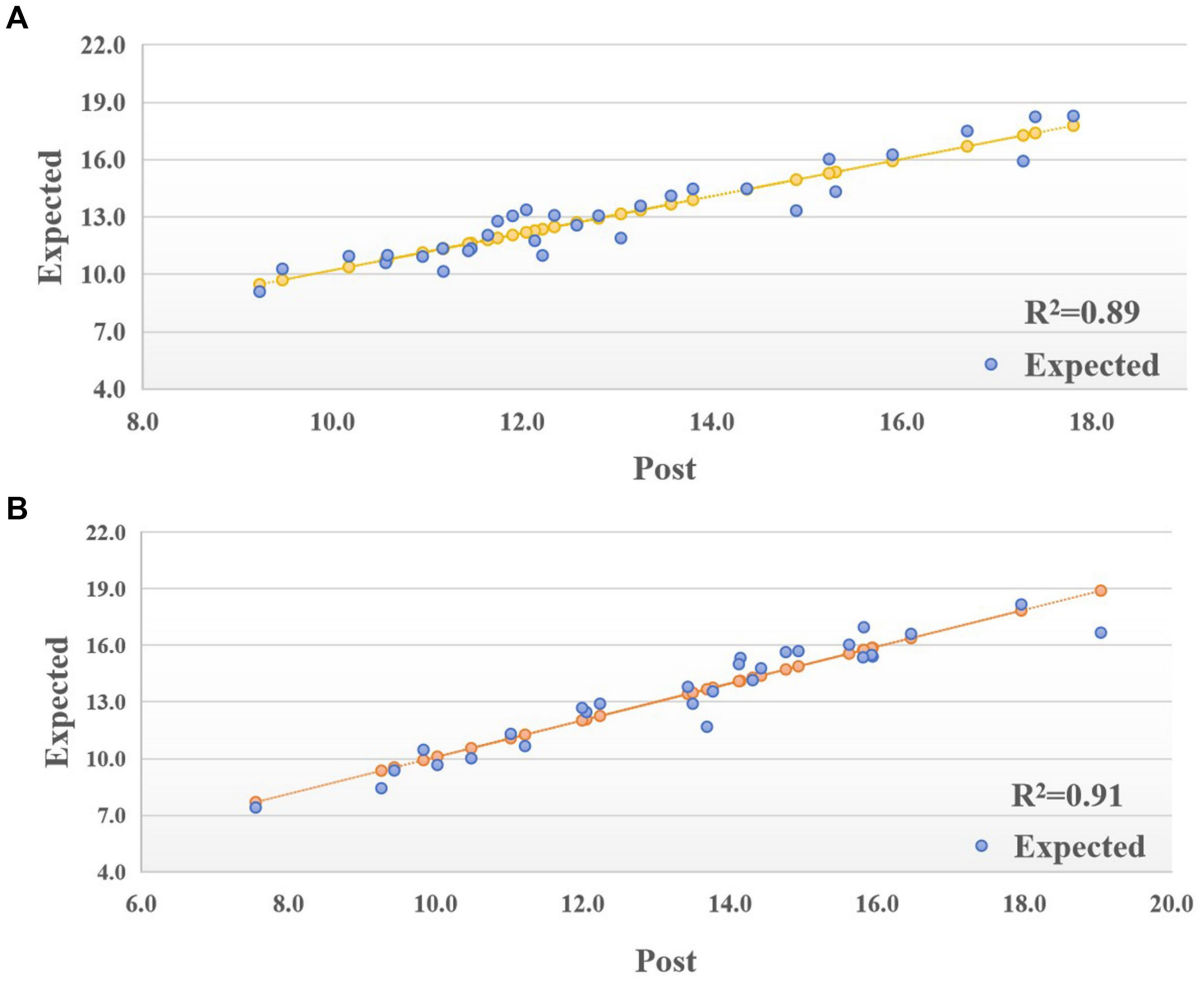
**(A)** Linear Regression of post-E-VO_2_peak and expected-E-VO_2_peak in female. **(B)** Linear Regression of post-E-VO_2_peak and expected-E-VO_2_peak in male.

Linear regression confirmed strong correlation between expected-E-VO_2_peak and post-E-VO_2_peak improvements (R^2^ = 0.89 for females, R^2^ = 0.91 for males; *p* < 0.001), with non-zero slopes indicating robust predictive validity (Figures 10A,B).

**Figure 11:**
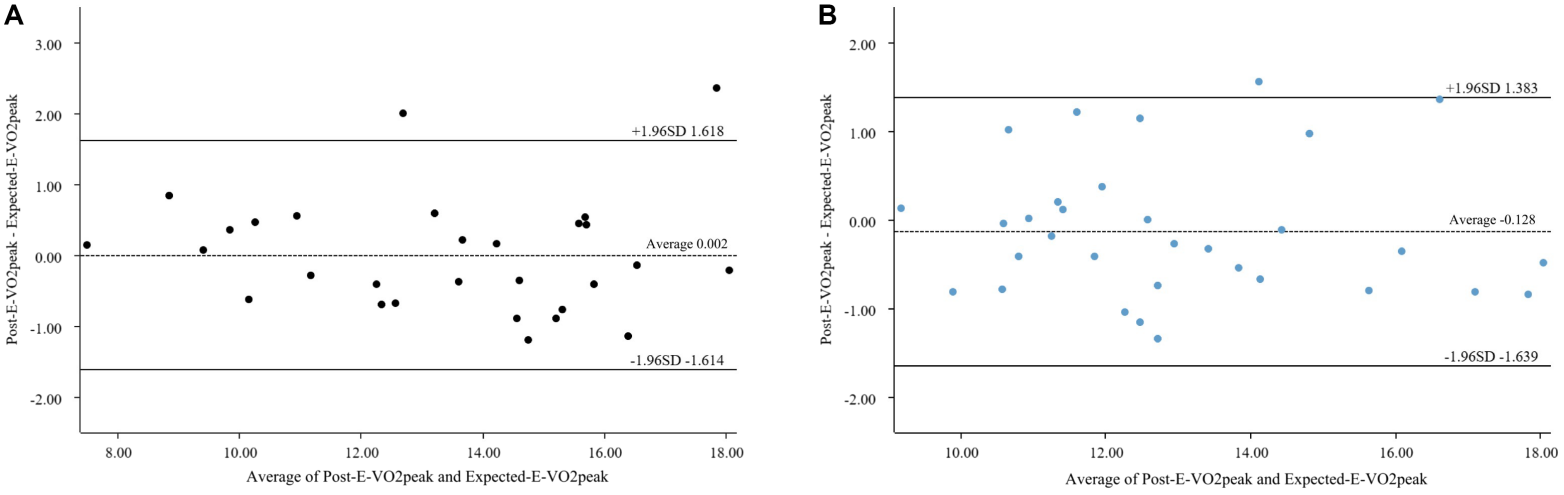
**(A)** Bland–Altman analysis of female’s CRF. **(B)** Bland–Altman analysis of male’s CRF.

Table 6 shows the “hit rate” of CRF improvement for 61 subjects within a range of one standard deviation and 1.96 times standard deviations (105, 106), with 70% (43/61) and 93% (57/61), indicating satisfactory CRF improvement across the board. To comprehensively assess model performance, we quantified the bias in predictions using a Bland–Altman plots (Figures 11A,B). The mean bias between expected and post-E-VO₂peak improvement was −0.128 mL/kg/min (95% LoA: −1.639 to 1.383) and 0.002 mL/kg/min (95% LoA: −1.614 to 1.618) for females and males, respectively, indicating that there was no systematic over/underestimation. Notably, 96.9 and 93.1% of the data points fell within the LoA range, respectively, which is consistent with hit rates of 91% and 97 in the 1.96-SD range, reinforcing the model’s precision across heterogeneous populations. The validity of the model in different populations was demonstrated by hit rate and Bland–Altman plot analyses.

**Table 6:** “Hit rate” of improvement at one standard deviation and 1.96 times standard deviation range.

Statistical range	Female		Male	
	Range	Hit rate	Range	Hit rate
M ± sd	3–17%	66%(21/32)	5–19%	76%(22/29)
M ± 1.96sd	−3–23%	91%(29/32)	−2–28%	97%(28/29)

## 4 Discussion

The study aims to develop a personalized exercise prescription for improving CRF in older adults through a scientifically robust framework. Conventional exercise prescriptions often adopt a one-size-fits-all approach, failing to account for the physiological heterogeneity inherent in older adults. To address this limitation, we employed a mixed-methods approach, integrating a systematic literature review with experimental validation to inform a BPNN model. This dual-source strategy leverages historical intervention patterns and real-world physiological variability, enabling the model to balance clinical feasibility with personalized adaptability. The proposed framework aims to optimize exercise prescriptions by dynamically aligning with individual functional baseline, ultimately enhancing CRF and supporting functional independence in older adults.

### 4.1 Accuracy of model predictive performance

Error metrics are crucial for assessing model performance. Our model demonstrated a more positive trend in terms of average RMSE and MAE (1.4383 vs. 1.5206) and (1.2366 vs. 1.5394) respectively, with an R^2^ value of more than 0.99. Like Parab et al., who achieved high accuracy in predicting blood urea via BPNN (RMSE of 0.69 and 2.06), our model leverages nonlinear mapping for CRF optimization (17). However, unlike their static health metrics, our framework incorporates dynamic exercise adaptation. Liu et al.’s (107) proposed a BPNN-based propagation delay prediction model showed the RMSE and MAE of 6.2457 and 5.0817, respectively, under optimal parameters. In comparison, our model showed more positive trend in prediction accuracy. Additionally, when compared to previous studies (95, 102, 103), it indicates that our BPNN model has better accuracy in predicting performance.

Traditional linear models fail to address the complex relationships between individual factors (e.g., age, BMI) and exercise prescription elements due to inherent physiological heterogeneity in older adults. While prior studies, such as Beltrame et al. (21) demonstrated NN’s utility in predicting aerobic energy expenditure (R = 0.98) using limited inputs (e.g., heart rate, speed), their frameworks lacked multidimensional older adults features. Our BPNN model addresses this gap through three key innovations: (i) Dynamic parameter integration: Unlike static models focusing on single parameters, our architecture normalizes age, sex, BMI and the initial value of VO_2_max to capture nonlinear interactions. For example, older adults with higher BMI required lower exercise intensity—a relationship detectable only through multilayer nonlinear mapping. (ii) LM optimization: The LM algorithm’s adaptive damping factor (*λ*) balanced convergence speed and stability, achieving smaller errors and higher predictive performance than other NN algorithms and traditional multiple linear regression models (108), enabling efficient handling of medium-scale datasets (N = 1,594) without sacrificing precision. (iii) Data augmentation: By expanding age, BMI and VO_2_max via MATLAB’s Randn function, we mitigated overfitting while preserving physiological plausibility. These design choices collectively yielded superior performance (RMSE = 1.44 vs. 3.032 in Dai et al.; 6.77 in Lin et al.), with MSE = 4.5416 and near-perfect correlation coefficients (R > 0.99 across training, validation, and test sets). The results confirm the model’s ability to generate precise, individualized prescriptions for intensity, time, and volume—critical for optimizing CRF in aging populations.

During the model prediction ability test, we observed minimal differences between the target output and the model-predicted exercise intensity, time and volume, with mean difference of 0.003 ± 0.099, −1.127 ± 6.094, and 0.314 ± 1.750, respectively. These findings, as well as mean error ratios of these elements that were mostly below 20%, mean that our BPNN model accurately predicted the exercise prescription elements within an acceptable margin of error. Importantly, when combining these metrics with clinical guidelines, the error ratio for exercise intensity (7% ± 12%) remained within the ACSM-recommended target range for aerobic exercise of 50–85% HRR (14). Similarly, the error ratio in exercise time (−5% ± 9%) was consistent with the guideline of “30–60 min of moderate-intensity activity per session,” and the error ratio for exercise volume (−7% ± 14%) was consistent with intervention periods that have been shown to be effective in improving CRF in older adults (e.g., 12 weeks) (109). These results suggest that even with prediction error, model-generated exercise prescriptions remain within the range of clinical safety and effectiveness.

Our BPNN model is accurate and precise in predicting various elements of exercise prescription for older adults, with error results for each element falling within the acceptable range. This provides an important reference and support for the development and experimental validation of exercise prescription tailored to older adults.

### 4.2 Validation of model in experimental validation

After the experimental validation intervention, the female group showed an 8.9% CRF improvement rate, and the male group showed a 10.0% CRF improvement rate, with both groups showing positive changes consistent with the model’s predictions. Interestingly, the wider improvement range in males (8.9–15.8% vs. 9.2–10% in females) may reflect higher baseline muscle mass and testosterone levels, which enhance CRF adaptation (110). However, females exhibited more consistent gains, possibly due to better adherence to low-intensity prescriptions—a pattern also observed in the FIT-Aging trail (111). This outcome lends support to the validity of our prediction model. The unique feature of our prediction model is its individualized consideration. We know that every individual’s health is distinct, as it is influenced by variables such as age, sex, BMI, and CRF level. These elements impact a person’s ability to exercise effectively and safely. Therefore, we personalize each subject’s exercise prescription based on this information. Meanwhile, combining our model with wearable devices (e.g., Polar H10) enables real-time intensity adjustment. For example, during a workout, if the heart rate deviates outside the margin of error, the system automatically suggests decreasing/ increasing elastic band resistance. This helps not only to ensure the safety of the subject, but also to improve the effectiveness of the exercise, which in turn improves the subject’s health.

We further validated the quantitative relationship between the expected-E-VO₂peak predicted by the model and the measured post-E-VO₂peak by linear regression analysis. The regression results for the female group showed that the model predictions explained 89% of the variance in CRF improvement (R^2^ = 0.89, 95 CI: 0.84–0.93, *p* < 0.001), and the explanatory power was even higher in the male group at 91% (R^2^ = 0.91, 95% CI: 0.87–0.95, *p* < 0.001; Figures 10A,B). Despite the BPNN’s inherent ability to model nonlinearly, verification of the linear consistency of its outputs with actual values through linear regression doubly confirms the biological plausibility of the model. Compared with traditional multiple linear regression models (based on static parameters such as heart rate, weight and height, R^2^ = 0.702) (112), the prediction of dynamic indicators of CRF in this model is more challenging, but by incorporating the dynamic parameters of exercise prescription (intensity, time) and the multilayer nonlinear fitting capability of the BPNN still achieves a high degree of accuracy, which significantly enhances the explanatory power of prediction (ΔR^2^ ≈ 0.2) and is more relevant to the the actual needs of chronic disease management.

With respect to the “hit rate” of CRF improvement, 70% of the subjects (43/61) achieved improvement within one standard deviation, while 93% (57/61) saw improvement within 1.96 times the standard deviation, suggesting that the model is satisfactory for overall CRF improvement.

To rigorously validate the consistency of the predictions, we used a Brand-Altman analysis (Figures 11A,B). In general, it is considered that the points in the graph should be located within the Lo A (mean ± 1.96 times standard deviation) range for 95% of all points, and it is also important to consider that the Lo A is not outside the range of professionally acceptable thresholds, and these requirements are generally considered to be a better consistency between the two methods (101). The results showed that the mean difference between predicted and observed improvements in VO₂max was −0.128 mL/kg/min (95% LoA: −1.2 to +1.5) and 0.002 mL/kg/min (95% LoA: −1.614 to 1.618) for females and males, respectively, with 96.9 and 93.1% of data points within the range of agreement. This is consistent with a 93% hit rate in the 1.96-SD range, demonstrating that the model avoids systematic bias while capturing interindividual variability. In contrast, the noninvasive blood pressure prediction model reported a wider LoA (−6.349 to 6.361 mmHg) (113), reflecting the increased complexity of the CRF dynamic metrics relative to static weight metrics.

Compared with models predicting static metrics such as weight change (114), the performance of the models in this study was superior. For example, the weight prediction model had a wider range of consistency bounds (−2.5 kg to 3.1 kg) and a Mean Absolute Percentage Error (MAPE) was 3.5%. Whereas CRF, as a dynamic physiological indicator in this study, has a higher prediction complexity, the present model demonstrated its robustness in the prediction of complex physiological adaptations through tighter error control (94% of data points were locate within the consistency boundaries) and higher explanatory power (R^2^ > 0.9).

### 4.3 Strengths and limitations

It is important to note that our BPNN model may be accurate and precise for predicting various elements of the exercise prescription for older adults with characteristics like those participants examined in this study. It is unclear if similar findings would be observed for individuals who may have different characteristics such as the presence of additional chronic health conditions or those with mobility limitations.

This study boasts several advantages. First, it generated a personalized exercise prescription model using BPNN to enhance CRF in older adults. The model’s predictions suggest that the method is feasible. Furthermore, the validity of the method is supported by the anticipated improvement in CRF following intervention based on model-generated exercise prescriptions for older adults.

The long-term goal of this model is to improve the physical fitness of older adults, especially their CRF and endurance levels. Through sustained personalized exercise interventions, we aim to bolster the CRF of older adults, thereby improving the efficiency of their cardiorespiratory system, reducing the risk of cardiovascular diseases, and decelerating age-related decline in muscle mass and bone density. The study confirmed that CRF levels were significantly associated with VO_2_max < 15 mL/kg/min, those who achieved 15–22 and > 22 mL/kg/min decreased their overall mortality risk ratio to 0.66 and 0.45, respectively (115). Further analyses suggest that CRF improvement not only reduces health risks but also translates into significant economic benefits. Assuming every 3.5 mL/kg/min increase in VO_2_max levels, normal-weight individuals save approximately $3,272 in healthcare costs annually (116). If extended to China’s 267 million aging population, the potential socio-economic impact would be extremely far-reaching—not only reducing the burden on the public health system, but also indirectly releasing the pressure on family care by enhancing the ability of the aged to live independently, creating a multi-dimensional social benefit.

We acknowledge certain limitations in our study. A primary factor contributing to the inaccurate prediction of exercise intensity is that the included studies were all designed with different exercise intensities for older adults, such as HRR%, VO_2_max%, and HRmax%. Unavoidably, this introduces some random errors during the harmonization process. As a result, the accuracy of exercise intensity predictions cannot match the overall accuracy of forecasting for exercise duration and volume.

In actual exercise interventions for senior adults, exercise intensity is not fixed but varies within a range. Likewise, while coding the exercise intensity set, we utilized the median value of exercise intensities used across studies. Therefore, the expected exercise intensities should be regarded as indicative median values, and a 5 to 10% hike or reduction can be used as anticipated exercise intensity range. In addition, the 6MWT can only estimate, rather than directly measure VO_2_max, and thus we could not accurately determine the subjects’ VO_2_max. Although we used the standard 6MWT to estimate VO_2_peak in our study, this method still has some error and uncertainty. Therefore, our results may be limited by this estimation method. When choosing the type of exercise, considering the safety and motivation of older adults, our experiment focused solely on elastic band training and excluded resistance exercise and high intensity interval training (HIIT), two exercise types that may improve CRF to some extent (117, 118). This specificity introduces two limitations: First, by not testing multiple modalities (e.g., cycling or swimming), our model’s generalizability is constrained. However, this reflects a deliberate trade-off given elastic bands’ advantage in accessibility, safety, and cost-effectiveness-critical for scalability in resource-limited communities. Second the absence of resistance exercise and HIIT prevents comprehensive assessment of different exercise types’ effects on CRF. Additionally, the lack of a comparison group using non-BPNN exercise prescriptions limits our ability to determine the model’s superiority over other methods. Future iterations could address these limitations by incorporating exercise type as an input variable.

Despite our model’ high level of simulation, we cannot yet generate completely individualized prescriptions for each person. For instance, the model would produce an identical prescription of exercise if two senior citizens have the same parameters. Hence, actual enhancements need to be verified in practice.

Going forward, we plan to increase the model’s sample size and implement a two-stage enhancement: (i) integrating Kalman filtering layers (99) between network modules to enable dynamic noise adaptation during real-time inference; specifically tackling motion-induced signal artifacts in heart rate monitoring during physical exercise; (ii) developing a hybrid preprocessing pipeline that combines the existing BPNN architecture with adaptive signal conditioning algorithms. These technical advancements will form the foundation for aged-targeted applications requiring robust handling of non-stationary physiological signals.

## 5 Conclusion

In this research, we suggest utilizing a model based on BPNN to develop individualized CRF exercise prescriptions for the older adults. This five-layer model, with a structure of 5–12–10-8-4, can generate tailored exercise prescription by processing the older adult’s basic information post-exercise evaluation and the targeted improvement degree of CRF. After augmenting the collected data, we develop an evaluation system to access cardiorespiratory health (VO_2_max) through machine learning and modeling, offering a convenient approach to health exercises for future older adult populations. Experimental results indicate a strong correlation between the predicted exercise prescriptions from our model and actual exercise prescriptions. To assess the model’s real-world improvement, we recruited subjects to participate in intervention based on the model-generated exercise prescriptions. The post-intervention VO_2_max closely matched the expected improved VO_2_max, confirming the high validity and reliability of our BPNN-based model for prescribing exercises to older adults.

This study provides initial evidence of the BPNN model in creating personalized exercise prescription for older adults, but more work is needed before this can be applied clinically broadly to older adults.

## Data Availability

The raw data supporting the conclusions of this article will be made available by the authors, without undue reservation.
